# ﻿A taxonomic revision of Thai *Fernandoa* Welw. ex Seem. (Bignoniaceae)

**DOI:** 10.3897/phytokeys.235.112839

**Published:** 2023-11-22

**Authors:** Chatchai Ngernsaengsaruay, Nattanon Meeprom, Weereesa Boonthasak, Yanatshara Attasook, Raweewan Thunthawanich

**Affiliations:** 1 Department of Botany, Faculty of Science, Kasetsart University, Chatuchak, Bangkok 10900, Thailand; 2 Biodiversity Center, Kasetsart University (BDCKU), Chatuchak, Bangkok 10900, Thailand; 3 Royal Botanic Garden Edinburgh, 20A Inverleith Row, Edinburgh, EH3 6LR, Scotland, UK

**Keywords:** Lamiales, morphology, palynology, second step lectotypification, Tecomeae, vegetative anatomy

## Abstract

A taxonomic revision of *Fernandoa* Welw. ex Seem. (Bignoniaceae) in Thailand is presented. Two species, *F.adenophylla* (Wall. ex G. Don) Steenis and *F.collignonii* (Dop) Steenis, are enumerated with updated morphological descriptions, illustrations and a taxonomic identification key, together with notes on distributions, distribution maps, habitats and ecology, phenology, conservation assessments, etymology, vernacular names, uses, and specimens examined. The collection of *Wallich Cat. 6502A* from Myanmar, Ava at G [G00133642] is designated here as the lectotype of *F.adenophylla* in a second step lectotypification. *F.collignonii* has a conservation status of Endangered [EN]. The leaf, stem, and wood anatomy and pollen morphology of *F.adenophylla* are also reported in this study.

## ﻿Introduction

*Fernandoa* Welw. ex Seem. is a small genus belonging to the tribe Tecomeae Endl. of the family Bignoniaceae (Fischer et al. 2004) and they are mostly trees occurring from tropical Africa to West Malesia. In total, the genus contains 15 species, of which five species dwell in Africa, three species in Madagascar (Bidgood 1994; Bidgood et al. 2006; POWO 2023) and seven species in Southeast Asia (Fischer et al. 2004; POWO 2023). These seven Southeast Asian species are *Fernandoaadenophylla* (Wall. ex G. Don) Steenis, *F.bracteata* (Dop) Steenis [native to Vietnam], *F.brilletii* (Dop) Steenis [native to Vietnam], *F.collignonii* (Dop) Steenis, *F.guangxiensis* D. D. Tao [native to Southern China], *F.macroloba* (Miq.) Steenis [Native to Sumatra] and *F.serrata* (Dop) Steenis [native to Vietnam] (POWO 2023). Seemann (1865: 330) when naming the new genus adopted Welwitsch’s recommendation and dedicated the genus to Ferdinand II (Portuguese: Dom Fernando II), King of Portugal (1816–1885), but the name was erroneously published by the printers as *Ferdinandia* Welw. ex Seem. Later, Seemann (1866: 123) corrected *Ferdinandia* to *Fernandoa* as Welsitsch intended. Subsequently, when Seemann (1870a: 280) named a new species from Africa, he treated *Fernandoa* as a typographical error and corrected it to *Ferdinandoa* Welw. ex Seem., in which he later reverted it to *Fernandoa* (Seemann 1871). Therefore, in botanical nomenclature, *Ferdinandia* and *Ferdinandoa* are orthographical variants (orth. var.) of *Fernandoa*. The Indomalesian genera, *Haplophragma* Dop, *Spathodeopsis* Dop and *Hexaneurocarpon* Dop, and the Malagasy genus *Kigelianthe* Baill. were later synonymised under *Fernandoa*, otherwise an African genus when it was first established (van Steenis 1976).

Morphologically, *Fernandoa* is similar to *Radermachera* Zoll. & Moritzi and *Stereospermum* Cham. in having decussate leaves, leaf rachises not keeled above, leaflets less than seven pairs, and septum of the ovary flat and without pseudoseptum. *Fernandoa* differs from *Radermachera* and *Stereospermum* based on lower surface of the leaflets with hairy domatia, fruits with longitudinal ribs, septum flat (vs lower surface of the leaflets without hairy domatia, fruits without longitudinal ribs, septum terete in *Radermachera* and *Stereospermum*) (Santisuk 1987; Fischer et al. 2004).

In Thailand, a taxonomic revision of the genus *Fernandoa* was published by Santisuk (1987) and two species were recognized, *F.adenophylla* and *F.collignonii*. In this paper, we had extensively examined Thai specimens of *Fernandoa* in various local and international herbaria including digital herbarium repositories. As a result, we hereby provide a comprehensive update to species descriptions, habitats and vernacular names, in addition to phenological observations, uses, IUCN conservation status, illustrations and distribution maps in Thailand for each species. Besides that, leaf, stem and wood anatomical characters and pollen morphology of *F.adenophylla* are presented, excluding *F.collignonii* because we did not collect specimens of this species.

## ﻿Materials and methods

Herbarium specimens deposited in BK, BKF, QBG, and those included in the digital herbarium databases of G (G-DC) (http://www.ville-ge.ch/musinfo/bd/cjb/chg/index.php?lang=en), K (including K-W) (http://www.kew.org/herbcat), L (https://bioportal.naturalis.nl/), P (https://science.mnhn.fr/institution/mnhn/collection/p/item/search), and US (https://collections.nmnh.si.edu/search/botany/) were examined (all herbaria acronyms follow Thiers 2023, continuously updated). The taxonomic history of *Fernandoa* was compiled using the taxonomic literature (Don 1838; de Candolle 1845; Seemann 1865, 1866, 1870a, 1870b, 1871; Kurz 1877; Clarke 1884; Ridley 1923; Dop 1925, 1930a, 1930b; Santisuk 1973, 1974, 1987; van Steenis 1976, 1977; Kochummen 1978; Santisuk and Vidal 1985) and online databases (IPNI 2023; POWO 2023). The morphological characteristics, distributions, ecology, and phenology were described from historic and newly collected herbarium specimens and the author’s observations during field work. The vernacular names were compiled from specimens examined and the literature (Santisuk 1974, 1987; Royal Institute 2013; Office of the Forest Herbarium, Forest and Plant Conservation Research Office, Department of National Parks, Wildlife and Plant Conservation 2014). The assessment of conservation status was performed following the IUCN Red List Categories and Criteria (IUCN Standards and Petitions Committee 2022) for a preliminary assessment of the conservation category in combination with GeoCAT analysis (Bachman et al. 2011) and field information. The calculation of Extent of Occurrence (EOO) and Area of Occupancy (AOO) are based on GeoCAT (https://www.kew.org/science/our-science/projects/geocat-geospatial-conservation-assessment-tool). The lamina, rachis, and stem (branch) anatomical characters of *Fernandoaadenophylla* were investigated by transverse sectioning with a sliding microtome at 15–20 μm thickness. For the study of epidermal cells of leaves, they were peeled and mounted. The wood samples of *F.adenophylla* were sectioned with a sliding microtome at 20–30 μm thickness along the transverse, tangential, and radial planes. The permanent slides of leaves, stems (branches) and wood were made following the standard methods of Johansen (1940) and Kermanee (2008). The anatomical characteristics were investigated and recorded photographically with an Olympus BX53 microscope and an Olympus DP74 microscope digital camera at the Department of Botany, Faculty of Science, Kasetsart University (KU). The anatomical terminologies follow those in the study by Metcalfe and Chalk (1957). The samples of pollen grains of *F.adenophylla* were examined and recorded photographically with an Olympus BX53 microscope and an Olympus DP74 microscope digital camera. Materials were prepared for scanning electron microscopy (SEM) at the Scientific Equipment Centre, Faculty of Science, KU by mounting pollen grains on stubs using double-sided sellotape, sputter-coating them with gold and examining them using an FEI Quanta 450 SEM (Hillsboro, OR, USA) at 15.00 KV. The characteristics of pollen grains were examined and measured, following Erdtman (1945, 1952) and Simpson (2010). The pollen morphology terminologies follow those of Punt et al. (2007).

## ﻿Results and discussion

### ﻿Taxonomic treatment

#### ﻿ Fernandoa

Taxon classificationPlantaeLamialesBignoniaceae

Welw. ex Seem., J. Bot. 3: 330. 1865 (sphal. Ferdinandia), nom. illeg.; J. Bot. 4: 123. 1866.

A044F906-E952-5373-80B4-8C968B532C75

Kigelianthe Baill., Hist. Pl. 10: 50. 1891.
Haplophragma Dop, Bull. Soc. Bot. France 72: 889. 1925.
Spathodeopsis Dop, Compt. Rend. Hebd. Séances Acad. Sci. 189: 1096. 1929; et Bull. Mus. Natl. Hist. Nat., Sér. 2, 2: 151. 1930.
Hexaneurocarpon Dop, Compt. Rend. Hebd. Séances Acad. Sci. 189: 1097. 1929; et Bull. Mus. Natl. Hist. Nat., Sér. 2, 2: 153. 1930.
Tisserantodendron Sillans, Bull. Soc. Bot. France 98: 270. 1952.

##### Type species.

*Fernandoasuperba* Welw. ex Seem. = *Fernandoaferdinandi* (Welw.) Baill. ex K. Schum.

##### Description.

Trees. ***Leaves*** 1-pinnate, imparipinnate, decussate; rachises not keeled above; leaflets 5–9, opposite, chartaceous to subcoriaceous, with scattered glands on both surfaces or a few scattered glands and small hairy domatia in the axil of lateral veins below. ***Inflorescence*** a terminal thyrse or raceme, densely stellate tomentose, densely dendroid tomentose, sparsely haired or glabrous. ***Flowers*** nocturnal; calyx persistent, campanulate or tubular-campanulate, irregularly 2–5-lobed; corolla yellowish-green, creamy white to pale yellow, corolla tube curved, constricted between basal and upper parts, basal tube short cylindrical, upper tube campanulate or infundibuliform-campanulate, bilabiate, 5-lobed, upper lobes 2 and lower lobes 3, subequal or unequal, crisped; stamens 4, didynamous, subexserted, anthers divaricate; staminode present; disc annular, surrounding the base of ovary; ovary superior, cylindrical, densely dendroid tomentose or glabrous, 2-celled, septum of the ovary flat without pseudoseptum, ovule numerous, axile placenta, style slender, stigma 2-lobed. ***Fruit*** a loculicidal capsule, cylindrical, twisted or straight to slightly arcuate, with longitudinal ridges, densely dendroid tomentose or glabrous, septum flat. ***Seeds*** numerous, flat, rather rectangular with a lateral hyaline-membranous wing.

A genus of fifteen species, distributed from Africa (5), Madagascar (3), and continental Southeast Asia to Sumatra (7); two species in Thailand.

### ﻿A key to the species of *Fernandoa* in Thailand

**Table:** 

1	Leaflets densely stellate and dendroid tomentose along midrib and basal part of lateral veins above, densely stellate and dendroid tomentose below; lowest pair of leaflets much reduced, resembling foliaceous pseudostipules; inflorescences (peduncles, axes and pedicels) densely stellate and dendroid tomentose; calyx campanulate, with 5 subequal or unequal lobes; upper tube of corolla campanulate; fruits twisted, with (6–)10 prominent longitudinal ridges; calyx, corolla, ovary and fruits densely dendroid tomentose	**1. *F.adenophylla***
–	Leaflets glabrous on both surfaces; lowest pair of leaflets not reduced to foliaceous pseudostipules; inflorescences (peduncles, axes and pedicels) sparsely hairy or glabrous; calyx tubular-campanulate, with 2–3 unequal lobes; upper tube of corolla infundibuliform-campanulate; fruits straight to slightly arcuate, with 6 prominent longitudinal ridges; calyx, corolla, ovary and fruits glabrous	**2. *F.collignonii***

#### ﻿1. Fernandoa adenophylla

**Figure 1.**
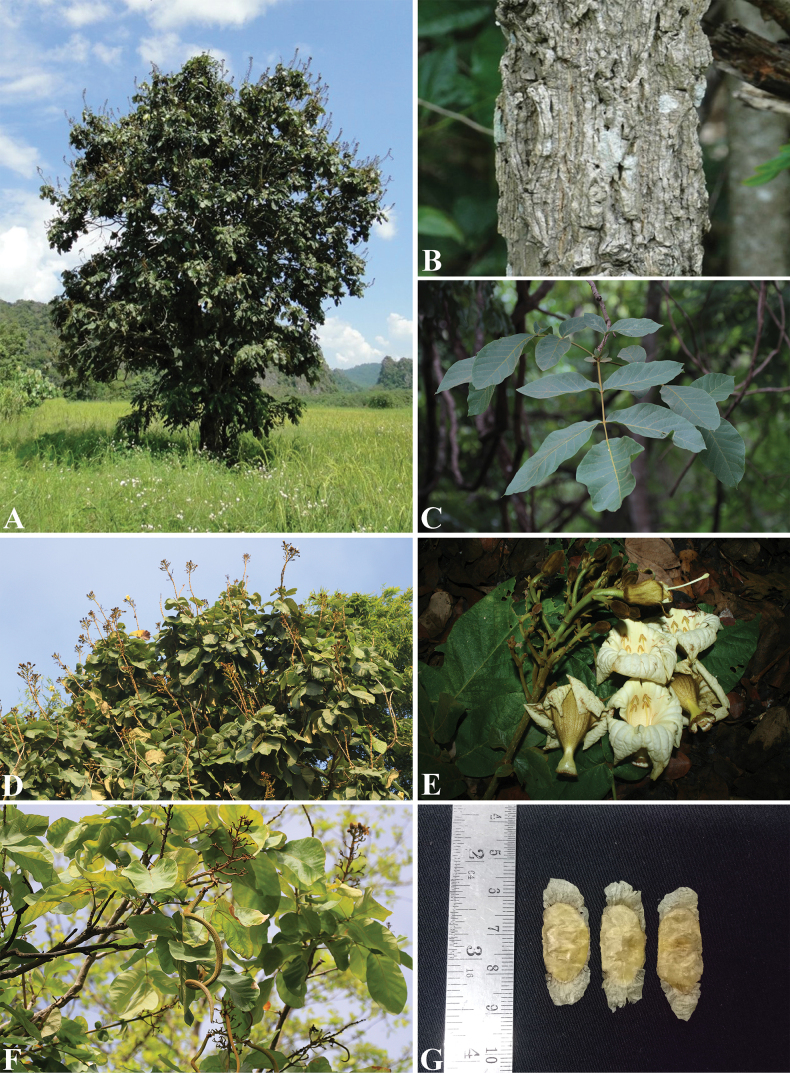
*Fernandoaadenophylla***A** habitat and habit **B** bark **C** branch and leaves **D** branches, leaves and inflorescences **E** inflorescences and flowers **F** branches, leaves, inflorescences and fruits **G** winged seeds. Photos: Yanatshara Attasook (**A**, **B**) Chatchai Ngernsaengsaruay (**C**–**G**).

Taxon classificationPlantaeLamialesBignoniaceae

(Wall. ex G. Don) Steenis, Blumea 23(1): 135. 1976; et Fl. Males., Ser. 1, Spermat. 8(2): 158. 1977; Kochummen in Ng, Tree Fl. Malaya 3: 39. 1978; Santisuk & J. E. Vidal in J.-F. Leroy, Fl. Cambodge Laos Vietnam 22: 39. t. 5, fig. 1. 1985; Santisuk in Smitinand & K. Larsen, Fl. Thailand 5(1): 47. 1987.

88E218E6-C3E5-501A-A23C-364F81FA6BCD

Figs 1 , 2

≡ Bignoniaadenophylla Wall. [Numer. List: 221. *Wallich Cat. 6502*, nom. nud.] ex G. Don, A Gen. Hist. 4: 221. 1838.
≡ Spathodeaadenophylla (Wall. ex G. Don) DC., Prodr. 9: 206. 1845.
≡ Heterophragmaadenophyllum (Wall. ex G. Don) Seem., J. Bot. 8: 340. 1870.
≡ Haplophragmaadenophyllum (Wall. ex G. Don) Dop, Bull. Soc. Bot. France 72: 890. 1925.

##### Type.

Myanmar, Ava, 12 Oct 1826, *Wallich 6502A* (lectotype, first step designated by van Steenis (1976: 135), G [without barcode], second step designated here G [G00133642, photo seen]; isolectotypes K-W [K001124064, photo seen], G [G00134691, G00134695, photos seen], P [P00609736, photo seen].

##### Description.

Trees deciduous, 5–15(–20) m tall, up to 170 cm girth; bark irregularly cracked, corky, grey to greyish-brown; young branches densely stellate and dendroid tomentose. ***Leaves*** decussate; petioles very short or up to 1.5 cm long; rachises 6–41 cm long, 4-angular, channelled above; petioles and rachises densely stellate and dendroid tomentose; leaflets 5–9, opposite, laminas variable in shape, size, apex and base, obovate, elliptic, ovate, oblong, suborbicular or orbicular, the terminal leaflets largest, 10.5–46.5 × 6.5–28 cm, the lateral leaflets 3–33 × 3–21 cm, the lowest pair of leaflets near the base of petiole much reduced, resembling foliaceous pseudostipules, 0.5–8.5 × 0.5–7 cm, apex obtuse, rounded, acute, acuminate, cuspidate or emarginate, base cuneate, oblique, obtuse, truncate, cordate or subcordate, margin entire or repand, subcoriaceous, glabrous above, except densely stellate and dendroid tomentose along midrib and basal part of lateral veins above, densely stellate and dendroid tomentose below, with scattered glands on both surfaces, midrib and lateral veins raised below, lateral veins 4–11 pairs, curving and connected in loops near the margin, veinlets reticulate, with small hairy domatia in the axil of lateral veins below; petiolules very short. ***Inflorescence*** a thyrse, 10–65 cm, erect, lax-flowered; peduncles 2–10 cm long; rachises 10–53 cm long; peduncles, axes and pedicels with dense stellate and dendroid tomentose. ***Flowers***: calyx yellowish-green, thick, 5-ribbed, persistent, densely yellowish-brown dendroid tomentose outside, glabrous inside, campanulate, 2–4.5 × 1.5–3 cm, bilabiate, 5-lobed, upper lobes (posterior) 3 and lower lobes (anterior) 2, subequal or unequal, lobes triangular, 0.7–1.8 × 0.4–1.7 cm, apex acute; corolla yellowish-green, creamy white to pale yellow, thick, densely yellowish-brown dendroid tomentose outside, glabrous inside, corolla tube curved, constricted between basal and upper parts, basal tube short cylindrical, widened towards the base, 1.5–2.5 cm long, 1–2.5 cm wide at base, upper tube widened towards the mouth, campanulate, 3–5.5 cm long, 3.5–5 cm wide at mouth, bilabiate, 5-lobed, upper lobes 2 and lower lobes 3, subequal or unequal, lobes suborbicular or broadly obovate, 2.5–4 × 2.4–5.5 cm, apex rounded, crisped; stamens 4, didynamous, subexserted, longer pair 3.2–5.5 cm long, shorter pair 3–4.5 cm long, filaments arcuate, creamy white to pale yellow, glabrous, anthers 5–9 mm long; staminode 1, needle-like, 0.7–2.2 cm long; disc annular, surrounding the base of ovary, creamy white to pale yellow; ovary cylindrical, 0.6–1.5 cm long, with (6–)10 longitudinal ridges, densely dendroid tomentose, style slender, 3–5 cm long, creamy white to pale yellow, glabrous, stigma 2-lobed, 4–7 mm long. ***Fruits*** cylindrical, 34–85 × 1.5–3.5 cm, green turning brown when dry, twisted, with (6–)10 prominent longitudinal ridges, densely yellowish-brown dendroid tomentose, septum 2–3 mm thick, 1–1.5 cm wide. ***Seeds*** flat, rather rectangular with a lateral hyaline-membranous wing, 1.5–4 × 0.6–1.4 cm.

**Figure 2.**
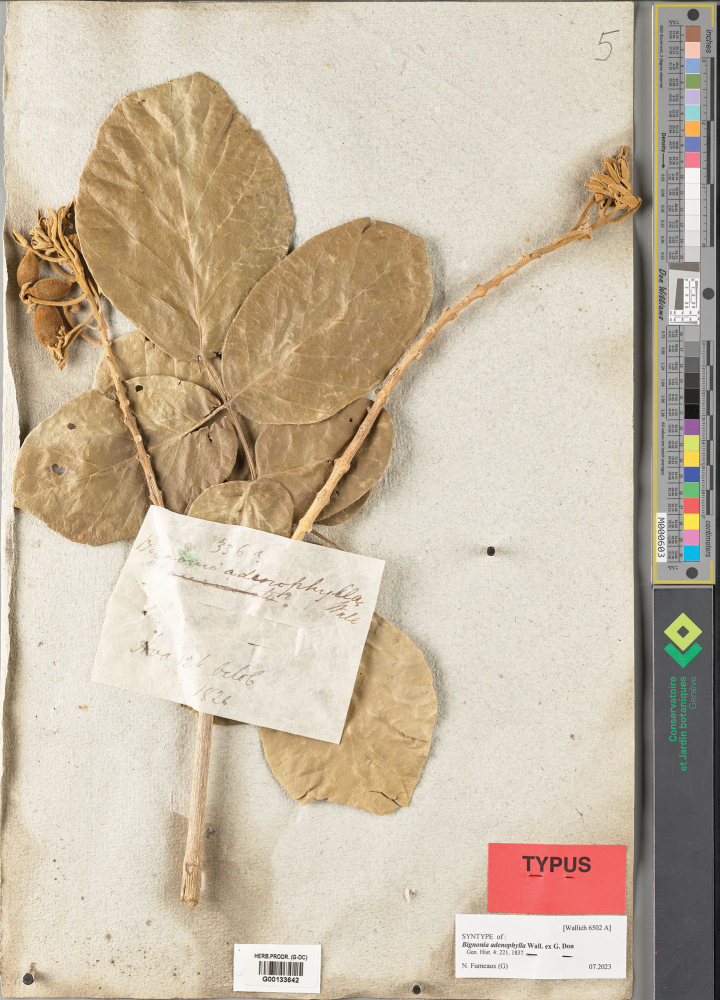
Lectotype of *Fernandoaadenophylla*, *Wallich 6502A* (G [G00133642]) from Ava, Myanmar. Photo: Conservatoire et Jardin botaniques de la Ville de Genève, Genève, Switzerland https://www.ville-ge.ch/musinfo/bd/cjb/chg/adetail.php?id=55790&lang=en.

##### Distribution.

India (Assam, Andaman and Nicobar Islands), Pakistan, Bangladesh, Myanmar, Vietnam, Laos, Cambodia, Thailand, Peninsular Malaysia.

##### Distribution in Thailand.

**Figure 3.**
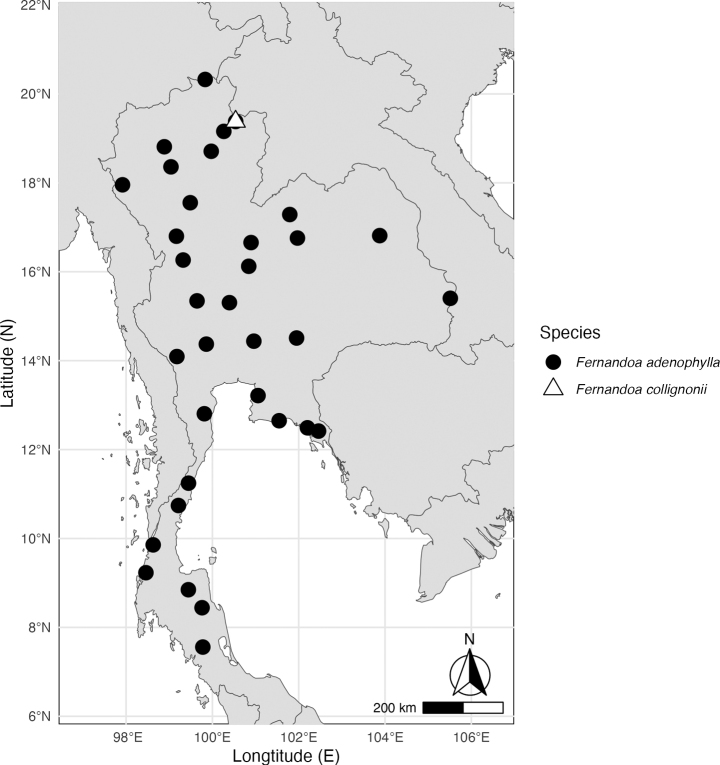
Distribution of *Fernandoa* in Thailand: *F.adenophylla* occurs in all floristic regions of Thailand and *F.collignonii* known only from Nan Province, Northern Thailand. [Thailand floristic regions follow *Flora of Thailand* Volume 16 Part 1 (The Forest Herbarium, Department of National Parks, Wildlife and Plant Conservation 2022)].

NORTHERN: Mae Hong Son, Chiang Mai, Chiang Rai, Nan, Lamphun, Lampang, Tak, Sukhothai, Phitsanulok, Kamphaeng Phet, Nakhon Sawan; NORTH-EASTERN: Phetchabun, Loei, Sakon Nakhon, Khon Kaen; EASTERN: Nakhon Ratchasima, Ubon Ratchathani; SOUTH-WESTERN: Uthai Thani, Kanchanaburi, Phetchaburi, Prachuap Khiri Khan; CENTRAL: Suphan Buri, Saraburi, Bangkok (Queen Sirikit Park, cultivated); SOUTH-EASTERN: Chon Buri, Rayong, Chanthaburi, Trat; PENINSULAR: Chumphon, Ranong, Surat Thani, Phangnga, Nakhon Si Thammarat, Trang. (Fig. 3)

##### Habitat and ecology.

It is found in deciduous dipterocarp and mixed deciduous forests (with or without bamboo), mixed deciduous forests with bamboo on limestone hills, savannas, gaps or edge of dry evergreen and lower montane rain forests, transition between deciduous and evergreen forests, secondary forests, disturbed open areas, along roadsides, along riverbanks, at elevations of near above mean sea level (a.m.s.l.) up to 1,000 m.

##### Phenology.

Flowering and fruiting nearly all year round.

##### Conservation status.

*Fernandoaadenophylla* is widely distributed from India to Indochina and Peninsular Malaysia, and has a large extent of occurrence (EOO of 3,353,755.49 km^2^) and area of occupancy (AOO of 352 km^2^). Also, considering it grows in secondary forests, disturbed open areas, and along roadsides, it is considered here as Least Concern (LC).

##### Etymology.

The specific epithet of *Fernandoaadenophylla* is derived from the Greek compound words, "aden-", "adeno-" meaning gland (glandular-), and “-*phylla*” meaning -leaved, refers to the leaves of this species with a few scattered glands on both surfaces (Stearn 1992; Radcliffe-Smith 1998; Gledhill 2002).

##### Vernacular name.

Khae khon (แคขน) (Northern); **Khae bit** (**แคบิด**) (Northern, North-Eastern); Khae phong (แคพอง) (Peninsular, Surat Thani); Khae rao (แคร้าว), Khae lao (แคลาว) (North-Eastern, Loei); Khae hua mu (แคหัวหมู) (Nakhon Ratchasima); Khae hang khang (แคหางค่าง) (General); Haeng pa (แฮงป่า) (Chanthaburi); Hong pa (โฮงป่า) (South-Western); Karen wood, Katsagon, Katsagon tree, Petthan (common name); Dhopa-paroli (Assam); Marodphali (Hindi).

##### Uses.

Flowers and young fruits are consumed as boiled or grilled vegetables and required cooking. Cultivated as shade and ornamental trees (the author’s observations). The wood is locally used in construction and used for making farming utensils. Bark, leaves, and seeds are used as medicinal purposes (Widodo 1998; Biodiversity-Based Economy Development Office (Public Organization) [BEDO] 2021).

##### Notes.

*Bignoniaadenophylla* was named by Wallich based on *Wallich Cat. 6502* collected from Myanmar: *6502A* from Irrawaddy River, Yenangheum (Yenangyaung), Prome, Sagaen (Sagaing), and Ava and *6502B* from Taong Dong but unpublished, and then this name was described by Don (1838: 221). van Steenis (1976: 135) mentioned *Wallich Cat. 6502* of Ava and Prome as type, when he transferred *B.adenophylla* under *Fernandoaadenophylla*. *Wallich Cat. 6502* represents two gatherings (two different materials collected from two different cites, which are distinguished by *Wallich Cat. 6502A* and *Wallich Cat. 6502B*, respectively). *Wallich Cat. 6502A* is from Ava and Prome and *Wallich Cat. 6502B* is from Taong Dong. Thus, *Wallich Cat. 6502A* could be regarded as the true type specimen, and *Wallich Cat. 6502B* from Taong Dong is not type.

van Steenis (1976) cited *Wallich Cat. 6502* from Ava and Prome at G as the holotype with isotypes in K and P. However, Wallich’s collection numbers are known to be curated by species generally from multiple collections (Noltie and Watson 2021); therefore, his erroneous designation of holotype effectively selected a) a collection for the lectotype if the collection consists of multiple sheets or b) the lectotype if it is a unicate gathering. van Steenis (1976) did not mention the number of specimens, and following Art. 9.6 of the ICN (Turland et al. 2018), they constitute syntypes. Therefore, the name *Fernandoaadenophylla* has been lectotypified in a first step by van Steenis (1976) using specimen *Wallich Cat. 6502A* at G [without barcode] with isolectotypes at K [without barcode] and P [without barcode]. We located three sheets of the specimen *Wallich 6502A* from Ava at G [G00133642, G00134691, and G00134695] and two sheets of the specimen *Wallich 6502A* from Prome at G [G00133632 and G00134708]; The G [G00133642] specimen is better preserved and more complete than the others, and hence is selected here in a second step lectotypification. We also traced isolectotypes at K-W [K001124064] and P [P00609736].

Santisuk (1987) reported that the leaves, calyx, and ovary have a stellate tomentum, but in this study the calyx, corolla, ovary, and fruits only exhibited dendroid trichomes, whereas the leaves bear both stellate and dendroid trichomes.

In addition to Santisuk (1987), the distribution in Mae Hong Son, Chiang Rai, Phayao, Nan, Lamphun, Sukhothai, Phitsanulok, Kamphaeng Phet, Nakhon Sawan, Sakon Nakhon, Khon Kaen, Ubon Ratchathani, Uthai Thani, Suphan Buri, Saraburi, Bangkok (cultivated), Rayong, Ranong, and Nakhon Si Thammarat Provinces were newly recorded in this study.

Santisuk (1987) reported that this species occurs in deciduous forests, savannas, and lowland secondary forests. Further, we confirmed it grows in more variable habitats (see habitat).

##### Additional specimens examined.

**Thailand. Northern**: Mae Hong Son [Sop Moei, 12 Jul 2013, *Pongamornkul 3468* (QBG); Sop Moei, 1 Jan 2014, *Pongamornkul 3928* (QBG); Ban Mueang Paeng, Mueang Paeng Subdistrict, Pai District; Salawin Forest Plantation, Mae Sariang District (Ngernsaengsaruay own observation)]; Chiang Mai [Locality not specified, 4 Oct 1921, *Nai Noi Mao s.n.* (BK) (Santisuk 1974); Chiang Dao District, fr., 27 Nov 1963, *Bunchuai 1375* (BKF); Mae Rim District, Mae Sa valley, in cultivation area, 1,000 m alt., fl., 15 May 1974, *Jackson 6039* (BKF); Hang Dong District, along the dirt road from Mae Heeyah Nai to Ban Bong, south side of Doi Suthep-Pui National Park, 425 m alt., fl., 15 Jul 1989, *Maxwell 89-903* (L [L3730390]); Doi Chiang Dao, SE foothills near Ban Yang Pong Luang, Chiang Dao District, 525 m alt., fl., 29 Oct 1989, *Maxwell 89-1326* (L [L3730538]); Doi Suthep-Pui National Park, Mae Rim District, in open place near Mae Sa stream, 650 m alt., fr., 9 Jan 1990, *Maxwell 90-37* (L [L3730536, L3730537]); Department of Biology, Faculty of Science, Chiang Mai University, 350 m alt., fl., 27 Jun 1992, *Palee 45* (L [L3730324]); Queen Sirikit Botanic Garden, Mae Rim District, 10 Aug 1994, *BGO. Staff 1294* (QBG); Maesa Elephant Camp, Mae Rim District, 25 Aug 1994, *BGO. Staff 1454* (QBG); Huai Gayo village area, San Kamphaeng District, 675 m alt., fr., 10 Oct 1995, *Palee 325* (BKF, L [L3730483, L3730484]); Mae Rim District, 5 Nov 1997, *Watthana & Siriphum 29* (QBG); Doi Suthep, *Kerr 3262* (ABD, BM, K) (Santisuk 1974)]; Chiang Rai [Doi Tung, base of the east side at Bah Kah (Akha) village, Mae Fa Luang District, 500 m alt., fl., 21 Jul 2006, *Maxwell 06-494* (L [L3730659]); Mueang Chiang Rai District (*Ngernsaengsaruay own observation*)]; Phayao [Rom Yen Subsistrict, Chiang Kham District, 18 Jun 2013, *La-ongsri* et al. *2920* (QBG)]; Nan [Mueang Nan District, 14 Nov 2009, *Srithi 345* (QBG); Song Khwae District, 18 Oct 2019 (the authors own observation)]; Lamphun [Mae Tha District, 29 Jan 2010., *Romkham 132* (QBG)]; Lampang [Ngao, Mae Huat, 350 m alt., fl., 18 May 1954, *Smitinand 1579* (BKF)]; Tak [along Mae Ping River, Om Lu Rapids, fl., fr., 9 Dec 1920, *Rock 629* (US [US03206561, US03206562, US03206563]); Doi Pha Wo, trail from Nam Dip to Pang Luang, Mae Sot District, fl., 19 Dec 1920, *Rock 669* (US [US03206564]); between Palut and Nam Dip, on the trail from Raheng to Mae Sot, fl., 17 Dec 1920, *Rock 1080* (US [US03206556]); Lan Sang National Park, bamboo rich gallery forest along cascade, fr., 27 Dec 1974, *Geesink*, *Hiepko & Phengklai 7941* (L [L2815249]); Lan Sang National Park, 300 m alt., fl., 5 Aug 1997, *Setbubpa 19* (BKF); Ban Tak District, 16 Oct 2014, *Tanming 715* (QBG); Mae Kor, *Winit 410* (ABD, K) (Santisuk 1974)]; Sukhothai [Si Satchanalai National Park, Si Satchanalai District, fl., 27 Jul 2015, *Maknoi 7946* (BKF, QBG)]; Phitsanulok [Thung Salaeng Luang National Park, c. 80 km east of Phitsanulok, c. 500 m alt., fl., 25 Jul 1973, *Murata*, *Fukuoka & Phengklai T-17126* (BKF, L [L2815258])]; Kamphaeng Phet [Mae Wong National Park, Khlong Lan District, 20 Oct 2001, *Watthana 1528* (QBG); Wang Khuang Subdistrict, Phran Kratai District (*Ngernsaengsaruay own observation*)]; Nakhon Sawan [Takhli District, 30 Jan 2013, *Maknoi 5210* (QBG)]; **North-Eastern**: Phetchabun [Khao Hin Pakarang Chon Daen District, 24 Nov 2009, *Maknoi & Tanaros 3425* (QBG); Chon Daen District, *Vacharapong 320* (BK) (Santisuk 1974)]; Loei [Wang Saphung District, 300 m alt., fl., 28 Apr 1946, *Nakkarn 66* (BKF); Wang Saphung District, 300 m alt., fl., y. fr., 28 Nov 1957, *Bunpheng 1030* (BKF, K) (Santisuk 1974); Pak Chom District, fl., 2 Sep 1968, *Phengnaren & Smitinand* 583 (BKF); Pha Daeng, Wang Saphung District, *Suvarnakoses* 1327 (BKF, K) (Santisuk 1974)]; Sakon Nakhon [Phu Phan National Park (*Ngernsaengsaruay own observation*)]; Khon Kaen [Ban Na Chan, Phu Pha Man National Park, 477.5 m alt., fr., 25 Feb 2011, *Norsaengsri*, *Tathana & Lakert 7616* (BKF, QBG)]; **Eastern**: Nakhon Ratchasima [Wang Nam Khiao District, Sakaerat Environmental Research Station, 420 m alt., fl., 17 Jul 1967, *Damrongsak 115* (BKF); Wang Nam Khiao District, fr., Dec 1967, *Damrongsak 418* (BKF); Khao Phayom, Wang Nam Khiao District, 430 m alt., fr., 21 Dec 1967, *Anan 15* (BKF); Pak Thong Chai District, Lam Phra Phloeng Dam, 400 m alt., fr., 29 Jan 1983, *Koyama*, *Terao & Wongprasert T-33101* (BKF); Nong Ra Wiang Subdistrict, fl., 3 Oct 2000, *Garcia & Phengkhlai 385* (BKF); Ban Badan, Pak Thong Chai District, *Sono 8* (BKF) (Santisuk 1974); Khao Phayom, Wang Nam Khiao District, *Nalampoon 15* (BKF) (Santisuk 1974)]; Ubon Ratchathani [Khong Chiam District (Ngernsaengsaruay own observation)]; **South-Western**: Uthai Thani [Khao Pla Ra, Lan Sak District, 19 Sep 2015, *Tanming 892* (QBG); Mueang Ka Rung Subdistrict, Ban Rai District; Phai Khiao Subdistrict, Sawang Arom District (Ngernsaengsaruay own observation)]; Kanchanaburi [Khwae Noi River Basin, near Neeckey (N. Wangka), 150 m alt., fr., 1 May 1946, *Bloembergen & Kostermans 276* (BK, K, L [L2815284, L2815285], P [P02902332]); Khwae Noi River Basin, Kin Sai Yok, c. 120 km northwest of Kanchanaburi, along river bank, 100–150 m alt., fl., 2 Aug 1946, *Kostermans 1436* (L [L2815280, L2815281], US [US03206558]); Ban Kao, fl., 14 Nov 1961, *Larsen 8202* (L [L2815289]); Sai Yok District, sterile, 15 Dec 1961, *Larsen 8733* (L [L2815287, L2815288]); Sai Yok District, 150 m alt., fl., 23 Nov 1971, *van Beusekom* et al. *3907* (BKF, L [L2815283]); Huai Ban Kao, 750 m alt., fr., 9 Nov 1971, *van Beusekom* et al. *3576* (BKF, L [L2815277]); Forest Research and Demonstration Center, Chalae Subdistrict, Thong Pha Phum District, 700 m alt., fr., 11 Dec 1995, *van Welzen 2* (L [L2815250]); Bo Phloi, Lam I Su, fr., 13 Apr 2000, *Phengklai* et al. *12171*, *12172* (BKF); Wang Pho, Lum Sum Subdistrict, Sai Yok District, 50 m alt., fl., 1 Jul 2006, *Chongko 532* (L [L3731040, L3731041], QBG); Mahidol University, Kanchanaburi Campus, Sai Yok District, 21 Jul 2006, *Maxwell 06-494* (QBG); Thipsukhontharam Temple, Huai Krachao District, 14 Oct 2014, *Tanming 633* (QBG); Khao Thong, *Kerr 19627* (ABD, BK, BM) (Santisuk 1974)]; Phetchaburi [Khao Phanoen Thung, Kaeng Krachan National Park, 23 Aug 1998, *Sasirat 80* (QBG); Thung Luang, *Kerr 20638* (ABD, BK, BM) (Santisuk 1974)]; Prachuap Khiri Khan [Bang Saphan District, *Put 1383* (BK, BM) (Santisuk 1974)]; **Central**: Suphan Buri [Phu Hang Nak, U Thong District (Ngernsaengsaruay own observation)]; Saraburi [Sam Lan Forest, Mueang Saraburi District, 75 m alt., fl., 27 Jul 1975, *Maxwell 75-722* (L[L2815259])]; Bangkok [Queen Sirikit Park, cultivated (Ngernsaengsaruay own observation)]; **South-Eastern**: Chon Buri [Si Racha District, sterile, 1 Dec 1927, *Collins 1748* (BK, US [US03206557]); Khao Khiao, Si Racha District, 100 m alt., fl., 13 Jun 1976, *Maxwell 76-393* (L [L2815252]); Khao Khiao Open Zoo, 9 Aug 2000, *Phengklai 12610* (BKF); Si Racha District, *Collins 223* (ABD) (Santisuk 1974)]; Rayong [Klaeng District, 19 Dec 2007, *Wessumritt 55* (QBG); Chanthaburi [Khao Sa Bap, Makham District, 11 May 1956, *Chit 333* (BKF) (Santisuk 1974)]; Trat [wayside near sea level, fl., 1 Aug 1973, *Murata*, *Fukuoka & Phengklai T-17348* (BKF, L [L2815254, L2815255], *Murata*, *Fukuoka & Phengklai T-17389* (BKF, L [L2815251])]; **Peninsular**: Chumphon [Tha Sae District, *Jaray 110* (BK) (Santisuk 1974); Kuring, *Kerr 11609* (BK, K, L [L2815292]) (Santisuk 1974)]; Ranong [30–70 km south of Ranong, 50–100 m alt., fl., 27 Apr 1974, *Larsen & S. S. Larsen 33441* (BKF, L [L2815256])]; Surat Thani [Ban Na San District, 100 m alt., fl., 14 Oct 1957, *Thaworn 500* (BKF, K) (Santisuk 1974)]; Phangnga [Nang Yon, Takua Pa, District, *Kerr 17047* (ABD, BK, BM) (Santisuk 1974)]; Nakhon Si Thammarat [Khiri Wong, Lan Saka District, fl., 18 Feb 1962, *Ploenchit 1792* (BKF); Tha Sala District, Walailak University, 10 m alt., fl., 22 Dec 2006, *Pooma*, *Pattharahirantricin & Sirimongkol 6528* (BKF); Trang [Khao Chong, Na Yong District, 80 m alt., fl., 29 Sep 1965, *Bunnab 6* (BKF, L [L2815293]); Khao Chong, Na Yong District, 84 m alt., fl., y. fr., Sep 1965, *Bunnab 193* (BKF); Huai Yot, 1 May 1916. *Vanpruk 852* (BKF)].

#### ﻿2. Fernandoa collignonii

Taxon classificationPlantaeLamialesBignoniaceae

(Dop) Steenis, Blumea 23(1): 136. 1976; Santisuk & J. E. Vidal in J.-F. Leroy, Fl. Cambodge Laos Vietnam 22: 42. t. 5, fig. 5–8. 1985; Santisuk in Smitinand & K. Larsen, Fl. Thailand 5(1): 48. fig. 20. 1987.

A3FA5F06-D544-5C7B-8BDB-D1D71F527865

Figs 4 , 5

≡ Spathodeopsiscollignonii Dop, Bull. Mus. Natl. Hist. Nat., Ser. 2, 2: 152. 1930.

##### Type.

Vietnam, Tonkin, Hoa Binh, Jul 1929, *Collignon s.n.* (holotype, P [P00609742, photo seen]).

##### Description.

Trees, 5–12 (–20) m tall; bark irregularly cracked, corky, grey to greyish-brown; young branches glabrous. ***Leaves*** decussate; petioles 3.5–9 cm long; rachises 8.5–17.5 cm long, terete, channelled above; petioles and rachises sparsely hairy or glabrous; leaflets 7–9, opposite, laminas elliptic, elliptic-oblong, oblong or ovate, 6–17 × 2.5–7 cm, apex acuminate or caudate, base oblique, cuneate or obtuse, margin entire, chartaceous, glabrous on both surfaces, except small hairy domatia in the axil of lateral veins below, with a few scattered glands below, midrib and lateral veins raised below, lateral veins 4–10 pairs, curving and connected in loops near the margin, veinlets reticulate, the lowest pair smaller than the upper pair of leaflets, not reduced to foliaceous pseudostipules; petiolules very short or up to 4 mm long. ***Inflorescence*** a thyrse, 10–21 cm; peduncles 2–4 cm long; rachises 3.5–10 cm long; peduncles, axes and pedicels sparsely hairy or glabrous. ***Flowers***: calyx thick, persistent, in flower buds 5-ribbed at least in the upper half, glabrous on both sides (sparse hairs outside in flower buds), tubular-campanulate, 2–3.5 × 1.5–2 cm, 2–3-lobed, unequal, apex acute (the posterior side with 2–3 lobes, halfway or more split towards the anterior base); corolla creamy white to pale yellow, glabrous on both sides, corolla tube curved, constricted between basal tube and upper tube, basal tube short cylindrical, widened towards the base, c. 2 cm long, 1–1.5 cm wide at base, upper tube widened towards the mouth, infundibuliform-campanulate, 4–4.5 cm long, 3.5–4 cm wide at mouth, bilabiate, 5-lobed, upper lobes 2 and lower lobes 3, subequal or unequal, lobes suborbicular or broadly obovate, 2–2.5 × 2–2.6 cm, apex rounded, crisped; stamens 4, didynamous, subexserted, longer pair c. 5 cm long, shorter pair c. 3 cm long, filaments arcuate, glabrous, anthers c. 6 mm long; staminode 1, needle-like, 5–6 mm long; disc annular, surrounding the base of ovary, c. 7 mm in diam.; ovary cylindrical, with 6 longitudinal ridges, glabrous, style slender, c. 4 cm long, glabrous, stigma 2-lobed. ***Fruits*** cylindrical, 33–70 × 3–6.5 cm, green turning brown when dry, straight to slightly arcuate, with 6 prominent longitudinal ridges, glabrous, septum 3–4 mm thick, 1.8–2.5 cm wide. ***Seeds*** flat, rather rectangular with a lateral hyaline-membranous wing, 4–4.5 × 1–1.8 cm.

**Figure 4.**
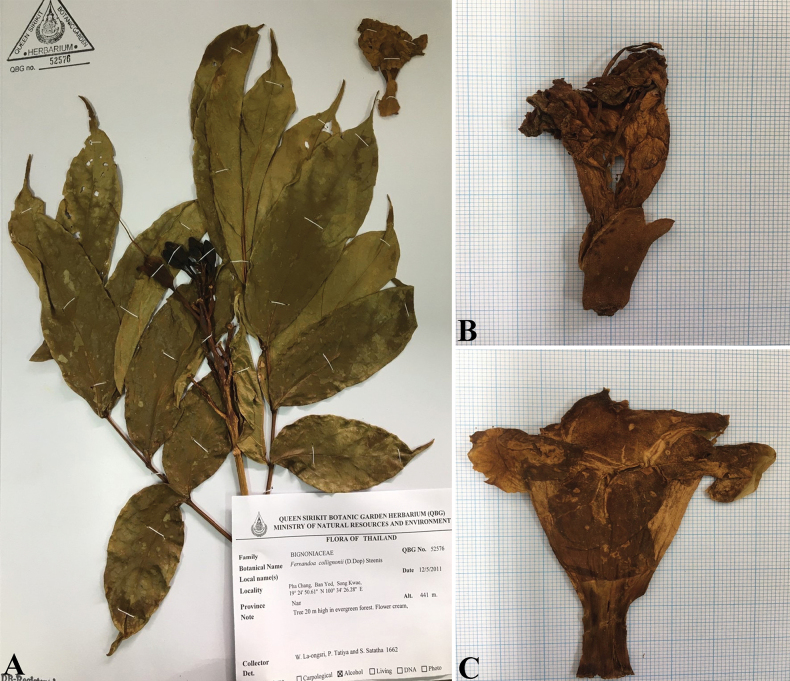
*Fernandoacollignonii***A** branch, leaves, inflorescence and flowers [*La-ongsri*, *Tatiya & Satatha 1662* (QBG)] **B** flower **C** corolla [*Srisanga*, *Maknoi*, *Panyachan & Tatiya 2884* (QBG)]. Photos: Pattarin Nunthamontree.

**Figure 5.**
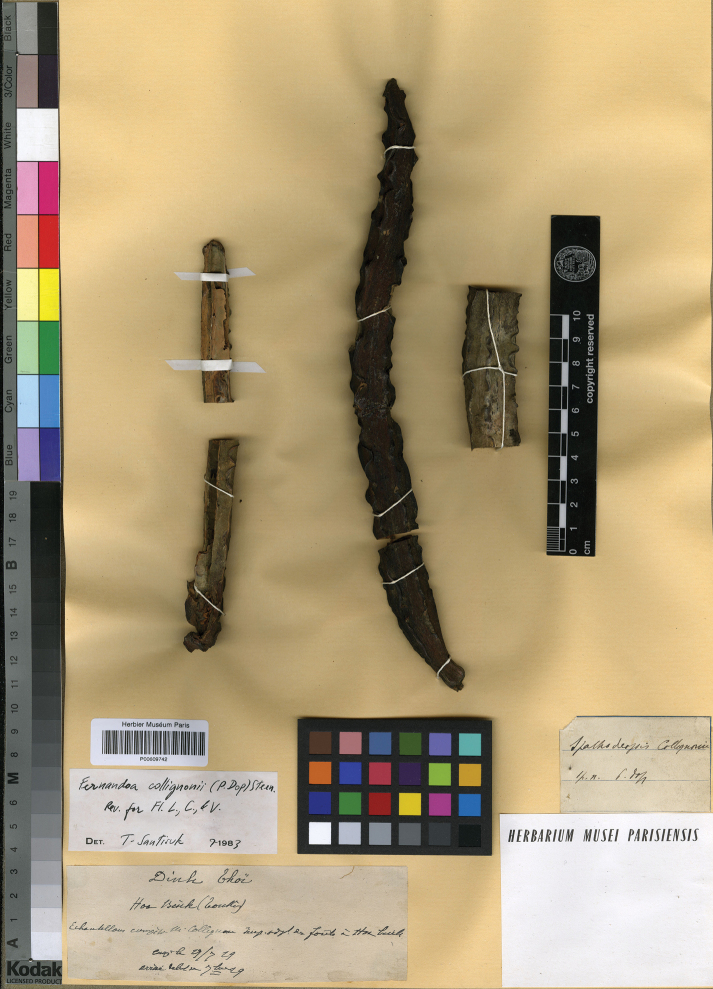
Holotype of *Fernandoacollignonii*, *Collignon s.n.* P [P00609742] from Hoa Binh, Tonkin, Vietnam, with immature fruits. Photo: Muséum National d’Histoire Naturelle (MNHN), Paris, France http://coldb.mnhn.fr/catalognumber/mnhn/p/p00609742.

##### Distribution.

Vietnam, Laos, Thailand.

##### Distribution in Thailand.

Northern: Nan. (Fig. 3).

##### Habitat and ecology.

It is found in dry evergreen forest, limestone hills, lower montane rain forest, at elevations of 400–800 m a.m.s.l.

##### Phenology.

Flowering April to July; fruiting July to December.

##### Conservation status.

Endangered (EN) (Santisuk et al. 2006; Chamchumroon et al. 2017). This species is known only from Indochina (Vietnam, Laos, and Thailand), and has a small extent of occurrence (EOO of 14,250.11 km^2^) and area of occupancy (AOO of 20 km^2^). In Thailand it is known only from Northern Thailand, Nan Province, and has a small extent of occurrence (EOO of 1,378.75 km^2^) and area of occupancy (AOO of 16 km^2^). It is appropriate to consider its status as Endangered [EN B2ab(ii, iv)].

##### Etymology.

The specific epithet of *Fernandoacollignonii* honours L. Collignon, the collector of the type specimen.

##### Vernacular name.

Khae dok lueang (แคดอกเหลือง) (Nan) [*Niyomdham & Puudjaa 7677* (BKF)]; **Khae hang khang san** (แคหางค่างสั้น) (Northern); Dinh thoi (Tonkin); Dinh, Dinh vang, Dinh collignon (Vietnam).

##### Uses.

No data recorded in Thailand. In Vietnam, Tonkin, Hoa Binh, the specimen Poilane 13012 (P [P02862885]) noted that its timber is good for all purposes, not being attacked by termites.

##### Notes.

In addition to the key to the species, *Fernandoacollignonii* differs from *F.adenophylla* in its petioles 3.5–9 cm long (vs very short or up to 1.5 cm long because the lowest pair of leaflets near the base of petiole much reduced, resembling foliaceous pseudostipules), rachises terete (vs 4-angular), petioles and rachises sparsely hairy or glabrous (vs densely stellate and dendroid tomentose), the longest leaflets up to 17 cm (vs the longest terminal leaflets up to 46.5 cm and lateral leaflets up to 33 cm), leaflets chartaceous, with a few scattered glands below (vs subcoriaceous, with scattered glands on both surfaces).

Santisuk (1987) reported that the height of this species ranges from 5–12 m tall, but the specimens *Srisanga* et al. *2884* (QBG), *La-ongsri* et al. *1662* (QBG), and *La-ongsri* et al. *1869* (QBG) collected from Nan Province mentioned 20 m tall. *Poilane 13012* (P [P02862885]) collected from Vietnam was recorded to have trunk diameter to 1 m.

The flowers were mentioned by the specimens from Vietnam, *Evrard 515* (L [L2815229]) to be reddish orange (rouge ochre), and *Poilane 6055* (L [L2815228]) described them as white, but were recorded here as creamy white to pale yellow in this study.

Santisuk (1973, 1974) reported that this species is known only from Nan Province, noted as Mae Sanian, *Winit 1788* (BK, BKF, K). In addition, Tham Sakoen National Park and Pha Chang, Ban Yot, Yot Subdistrict, Song Khwae District, and Phu Huat, Sakat Subdistrict, Pua District were new localities recorded in this study.

Santisuk (1987) reported that this species is uncommon in evergreen forests. In addition, the specimen *Srisanga* et al. *2884* (QBG) was collected in dry evergreen forest, at elevation of 800 m a.m.s.l., the specimen *La-ongsri & Romkham 1311* (QBG) in limestone forest, and the specimen *Niyomdham & Puudjaa 7677* (BKF) in hill evergreen forest (= lower montane rain forest), at elevation of 800 m a.m.s.l. (see habitat).

##### Additional specimens examined.

**Thailand. Northern**: Nan [Sanian Subdistrict (noted Mae Sanian), in evergreen forest, 420 m alt., fr., 5 Aug 1926, *Winit 1788* (BK, BKF, K) (Santisuk 1973, 1974); Phu Huat, Sakat Subdistrict, Pua District, in hill evergreen forest, 800 m alt., fl., 19 May 2006, *Niyomdham & Puudjaa 7677* (BKF); Tham Sakoen National Park, Yot Subdistrict, Song Khwae District, in dry evergreen forest, 800 m alt., fl., 12 May 2006, *Srisanga* et al. *2884* (QBG); Tham Sakoen National Park, Yot Subdistrict, Song Khwae District, in limestone forest, noted that fruits were green, 16 Dec 2010, *La-ongsri & Romkham 1311* (QBG); Pha Chang, Ban Yot, Yot Subdistrict, Song Khwae District, in evergreen forest, 441 m alt., fl., 12 May 2011, *La-ongsri*, *Tatiya & Satatha 1662* (QBG); Tham Sakoen National Park, near stream in evergreen forest, 600 m alt., fr., 27 Jul 2011, *La-ongsri* et al. *1869* (QBG)].

**Vietnam** [Tonkin, Hoa Binh, 27 Aug 1926, *Poilane 13012* (P [P02862885]); Tonkin, Hoa Binh, fl., s.d., *Brillet 11* collected from the type locality (K [K000779292], P [P02862889]); Ninh Binh Province, Cuc Phuong National Park, noted with fruits, 16 Nov 2001, *Cuong 1548* (L [L3730433]); Annam, Nha Trang, noted that flowers white, 24 Apr 1923, *Poilane 6055* (L [L2815228]); Forêt sur le Song Cao de Song Trang à Binh Loi, près Nha Trang, fl., 16 Jul 1921, *Evrard 515* (L [L2815229]); Ninh Thuan Province, Ninh Hai District, Nui Chua National Park, fr., 16 Jan 2010, *Soejarto* et al. *DDS14712* (P [P03387600, P03387601])]; **LAOS** [Xieng Khouang, 8 Nov 1920. *Poilane 2309* (BKF, L [L3730861], P [P02862887]); s.d., *Spire 228* (P [P02862886])].

### ﻿Anatomical study

#### Leaves, stems (branches) and wood anatomy of *Fernandoaadenophylla*

**Figure 6.**
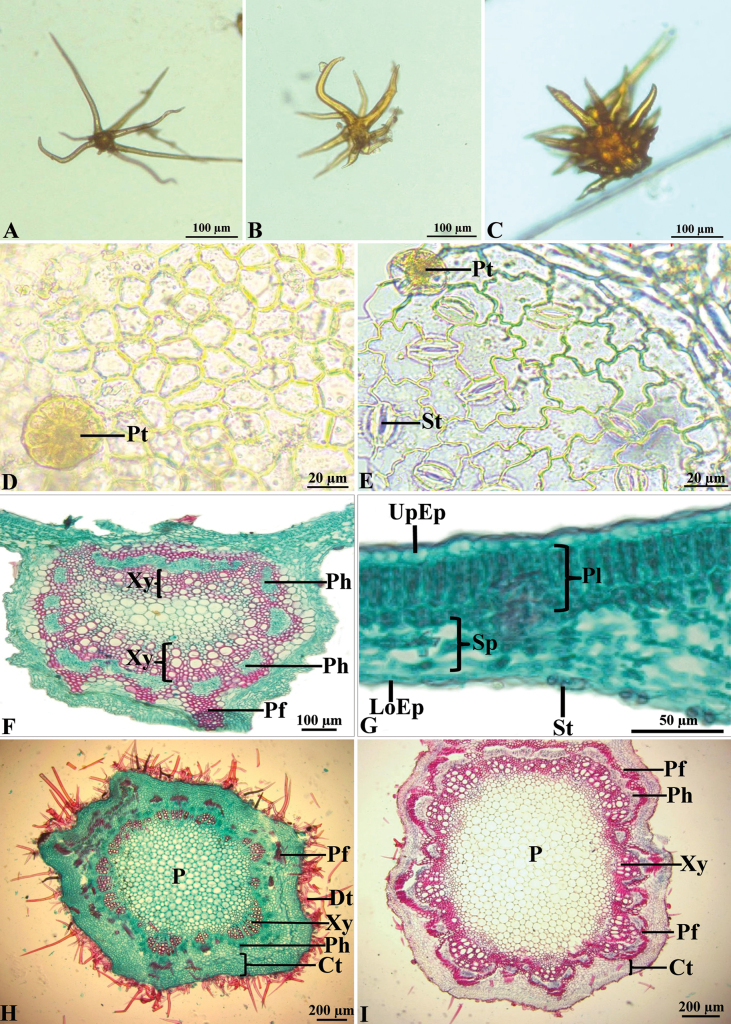
Leaf anatomy of *Fernandoaadenophylla***A** stellate trichome **B**, **C** dendroid trichome **D** upper epidermis **E** lower epidermis **F, G** transverse section middle of leaflet **H** transverse section middle of immature rachis **I** transverse section middle of mature rachis. [Ct = cortex, Dt = dendroid trichome, LoEp = lower epidermis, P = pith, Pf = phloem fiber, Ph = phloem, Pl = palisade cells, Pt = peltate glandular trichome, Sp = spongy cells, St = stomata, UpEp = upper epidermis, Xy = xylem], the stain combination safranine and fast green.

This species has branched eglandular trichomes, and are uniseriate, unicellular. The unicellular trichomes have only one cell but are quite variable in length. Branched eglandular trichomes can be divided into two types: stellate (star-shaped, with many branches radiating outwards) and dendroid (have a tree-like branching form). Both trichomes are also found on the petioles, rachises, and inflorescences (peduncles, axes, and pedicels), except on the calyx, corolla, ovary, and fruits where is only found a dendroid trichome. (Fig. 6A–C) Peltate glandular trichomes that are sunken into epidermal cells on both surfaces. The cuticular ornamentation is deposited on the outer wall of the epidermal cells. The epidermal cells are arranged in a single layer on both surfaces, and are larger on the upper surface than on the lower one. The epidermal cells on the upper surface are polygonal in shape with straight anticlinal walls, and on the lower surface are irregular in shape with undulate (wavy) anticlinal walls. The stomata are confined to the lower surface and are anomocytic. The mesophyll composed of palisade parenchyma (also called palisade mesophyll) underlying the upper epidermis and spongy parenchyma (also called spongy mesophyll) underlying the lower epidermis (bifacial leaf). The palisade parenchyma exhibits two layers: an upper tall one and a basal layer, about half in height, tightly packed cells and the spongy parenchyma comprised of loosely packed, irregularly shaped cells. In the midrib, the sclerenchymatous sheath of stele is made both by phloem fibers and lignified rays. Stele interpreted as two crescents, almost flat above and arched below, perhaps with two small bundles in the upper corners. The presence of sclerenchyma cells in the midrib is to provide support and protection for the leaf structure. (Fig. 6D–G).

The outline of the rachises in transverse section is 5-angular, it is channeled on the upper side. Stellate and dendroid trichomes are present as in the laminas. The epidermis in transverse section is circular or semicircular, and cells are usually smaller than cells in the ground tissue. The cortex of the young rachises is broader than the mature rachises. Parenchyma predominates in ground tissue, and fiber cells are present. The vascular bundles are completely ensheathed by sclerenchyma cells. The xylem is incompletely surrounded by phloem, interspaced with sclerenchyma cells. (Fig. 6H, I).

**Figure 7.**
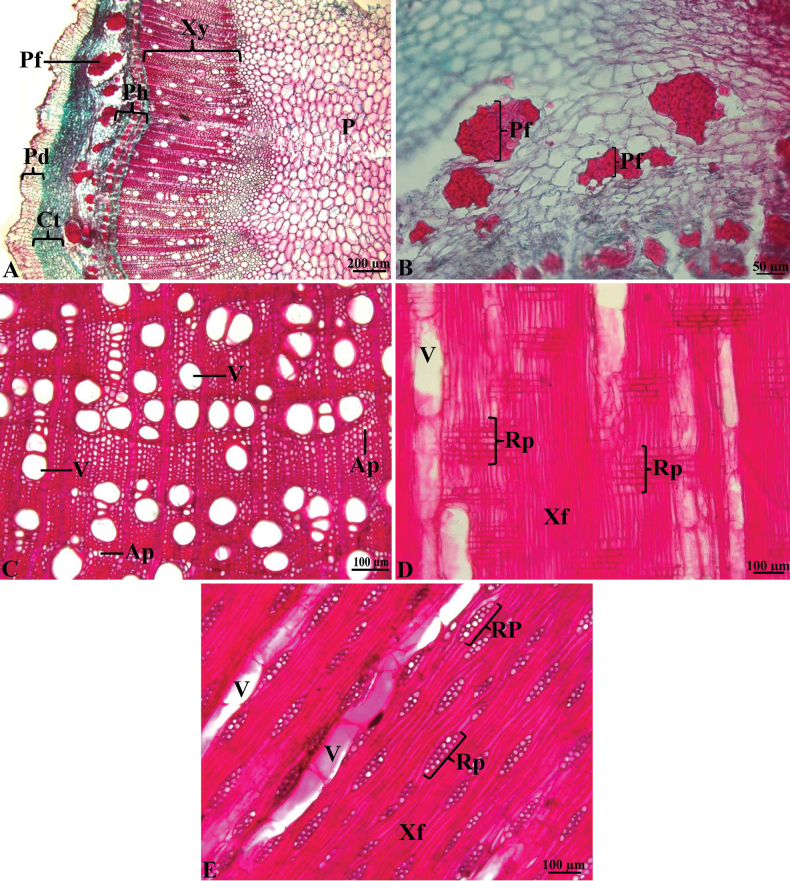
Stem (branch) and wood anatomy of *Fernandoaadenophylla***A, B** transverse section secondary growth of stem **C–E** wood anatomy **C** transverse section **D** radial longitudinal section **E** tangential longitudinal section (shown in an oblique orientation) [Ap = axial parenchyma, Ct = cortex, P = pith, Pd = periderm, Pf = phloem fiber, Ph = phloem, Rp = ray parenchyma, V = vessel, Xf = xylem fiber, Xy = xylem], the stain combination safranine and fast green.

Secondary growth of stems (branches): The bark is made up of the periderm (also called outer bark), the cortex, and the phloem (also called inner bark). The periderm is 7–10 layered. The cortical parenchyma is 7–8 layered. The cells of the vascular cambium divide and supply secondary phloem and xylem. Fibers occur both in the primary and the secondary phloem. The pith is only parenchymatous. (Fig. 7A, B).

*Fernandoaadenophylla* has diffuse-porous wood. Vessels (pores) are solitary and form in groups of 2–4 cells or more, 20–100 µm in diam. Vessel density ranges from 4–20 vessels per mm^2^. Axial parenchyma patterns are confluent. Rays are heterocellular, biseriate, sometimes uniseriate, with the procumbent cells 3–12 cells long, and with one row of the upright cells at both ends, and are sometimes homocellular with only the upright cells 2–3 cells long. The septate fibers are present. (Fig. 7C–E) The result of this study is consistent with Metcalfe and Chalk (1957) which reported wood anatomy of *Fernandoa*: rays are homocellular, sometimes heterocellular, with 4–11 cells long, and fibers are septate.

A comparison of wood anatomical characteristics of *Fernandoaadenophylla* with the previous studies of other two genera, *Dolichandrone* (Boonthasak and Ngernsaengsaruay 2021) and *Santisukia* (Meeprom et al. 2022) in the tribe Tecomeae of the family Bignoniaceae in Thailand is shown in Table 1.

**Table 1.** A comparison of wood anatomical characteristics of *Fernandoaadenophylla* with other two genera, *Dolichandrone* and *Santisukia* in the tribe Tecomeae of the family Bignoniaceae in Thailand.

Characters	*F.adenophylla*	*Dolichandrone*	*Santisukia*
Vessel arrangement	diffuse-porous	diffuse-porous	diffuse-porous
Vessel diameter (µm)	20–100	30–90	c. 100
Axial parenchyma	Confluent	banded, confluent	aliform, confluent
Ray parenchyma	biseriate, sometimes uniseriate heterocellular or uniseriate homocellular	uniseriate, sometimes biseriate heterocellular	biseriate, triseriate, tetraseriate heterocellular
Ray height (cells)	3–12	2–60	5–40
Ray width (rows of cells)	(1–)2	1(–2)	2–4
Fibers	Septate	septate	septate

### ﻿Palynological study

#### Pollen morphology of *Fernandoaadenophylla*

**Figure 8.**
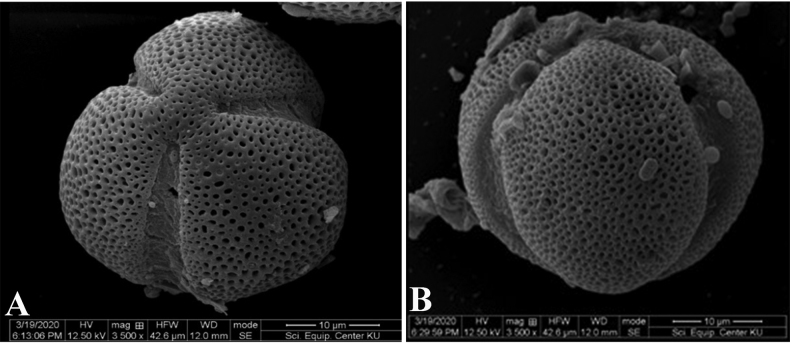
SEM micrographs of pollen grains of *Fernandoaadenophylla***A** polar view **B** equatorial view.

The pollen grains of *Fernandoaadenophylla* are monads, isopolar, tricolpate, oblate, suboblate to oblate-spheroidal in shape. The size of the pollen grains is medium to large, the polar axis ranges between 29–55 µm, and the equatorial axis ranges between 27–54 µm. The exine sculpturing is reticulate. (Fig. 8) Santanachote (1981) reported that the pollen grains of this species are suboblate in shape, the polar axis ranges between 41–51 µm, and the equatorial axis ranges between 35–41 µm which shows slight differences in shape and size from this study.

## Supplementary Material

XML Treatment for Fernandoa

XML Treatment for Fernandoa adenophylla

XML Treatment for Fernandoa collignonii

## ﻿Additional information

### Author ORCIDs

Chatchai Ngernsaengsaruay https://orcid.org/0000-0002-7131-976X

### Data availability

All of the data that support the findings of this study are available in the main text.
